# NADPH oxidase 4 as a source of hydrogen peroxide can regulate norepinephrine levels and vascular contraction

**DOI:** 10.3389/fendo.2026.1932927

**Published:** 2026-10-02

**Authors:** Heike Brendel, Jennifer Mittag, Nicole Bechmann, Amna Shahid Mehmood-Ashraf, Antje Schauer, Patrick Diaba-Nuhoho, Mirko Peitzsch, Graeme Eisenhofer, Stefan R. Bornstein, Henning Morawietz, Coy Brunssen

**Affiliations:** 1 Division of Vascular Endothelium and Microcirculation, Department of Medicine III, Faculty of Medicine and University Hospital Carl Gustav Carus at TUD Dresden University of Technology, Dresden, Germany; 2 Institute for Clinical Chemistry and Laboratory Medicine, University Hospital Carl Gustav Carus, Faculty of Medicine and University Hospital Carl Gustav Carus at TUD Dresden University of Technology, Dresden, Germany; 3 Department of Internal Medicine and Cardiology, Heart Center Dresden - Laboratory of Experimental and Molecular Cardiology, Technische Universität Dresden, Dresden, Germany; 4 Department of Medicine III, Faculty of Medicine and University Hospital Carl Gustav Carus at TUD Dresden University of Technology, Dresden, Germany; 5 Department of Environmental and Public Health, College of Health Sciences, Abu Dhabi University, Abu Dhabi, United Arab Emirates

**Keywords:** adrenal, adrenergic receptor, catecholamines, NADPH oxidase 4, vascular contraction

## Abstract

**Introduction:**

The adrenal glands play a pivotal role in maintaining physiological homeostasis, and disturbance in their function can lead to significant clinical disorders, including hypertension, and adrenal insufficiency. Reactive oxygen species (ROS), once considered merely byproducts of cellular metabolism, are now recognized as critical signaling molecules within the adrenal cortex and medulla. Among the major enzymatic sources of ROS are NADPH oxidases (Nox). Dysregulation of Nox-derived ROS has been linked to altered steroidogenesis, oxidative stress- mediated adrenal damage, and tumorigenic processes. We aimed to elucidate the role of Nox4 on adrenal function under healthy conditions.

**Methods:**

Using Nox4-knockout mouse model, adrenal effects were assessed by histological analysis, real-time PCR, and cathecholamine quantification using HPLC-ECD and LC-MS/MS. Cardiovascular function was evaluated by echocardiography, blood pressure measurements, and ex vivo vascular function analysis.

**Results:**

Here, we demonstrated that Nox4 is expressed in the mouse adrenal gland, and its deletion led to decreased hydrogen peroxide production, diminished dopamine beta-hydroxylase mRNA expression, and reduced norepinephrine levels. In chromaffin cells, treatment with hydrogen peroxide stimulated catecholamine release and increased dopamine beta-hydroxylase expression, indicating a regulatory role of hydrogen peroxide in catecholamine synthesis and secretion. Further analysis of the physiological consequences of reduced norepinephrine levels in Nox4^−/−^ mice revealed decreased alpha-1 adrenergic receptor expression and impaired adrenergic vascular contraction, while cardiac function remained unaffected. In a model of physical activity, Nox4 depletion resulted in smaller adrenal glands and lower urinary norepinephrine concentrations, further explaining diminished physical activity of Nox4^−/−^ mice.

**Conclusion:**

Our findings provide evidence that Nox4-derived H_2_O_2_ plays a role in norepinephrine release and norepinephrine-mediated functions. Hence, loss of Nox4 impairs vascular contractility and reduces physical activity performance, highlighting the physiological importance of Nox4 in adrenal function.

## 1 Introduction

The adrenal glands are small, triangular-shaped glands located on top of each kidney. They are critical components of the endocrine system, responsible for producing essential hormones, including cortisol, aldosterone, and catecholamines. These hormones regulate numerous functions, including stress responses, metabolism, immune responses, physical activity, and blood pressure (1). Proper adrenal function is vital for maintaining overall health and homeostasis, and the quality of life in patients with adrenal disease is markedly reduced (2).

The catecholamines dopamine, norepinephrine, and epinephrine are key neurotransmitters and hormones involved in stress response, mood regulation, and cardiovascular function. Catecholamine synthesis occurs mainly in the adrenal medulla and neurons of the sympathetic nervous system. In a first step, tyrosine is converted into l-DOPA by tyrosine hydroxylase, which is the rate-limiting step in the synthesis process. l-DOPA is then converted into dopamine by aromatic l-amino acid decarboxylase (AADC), followed by the conversion of dopamine into norepinephrine by dopamine beta-hydroxylase (DBH) and methylation of norepinephrine to epinephrine by phenylethanolamine *N*-methyltransferase (PNMT) (3). Norepinephrine and epinephrine are then stored in separate secretory vesicles. Upon sympathetic stimulation, these vesicles undergo Ca^2+^-dependent exocytosis, releasing catecholamines into the circulation (1). Catecholamine secretion from chromaffin vesicles can be triggered by various factors, including hypoxia, acidosis, hypoglycemia, and mediators such as histamine and angiotensin II (1).

Reactive oxygen species (ROS) have been implicated in several forms of adrenal diseases, such as excessive reactive oxygen species causing deleterious effects on adrenal function, leading to adrenal insufficiency (4). Reactive oxygen species are also recognized in the regulation of steroid hormones. Rajamohan et al. explored the role of ROS in angiotensin II (Ang II)-induced aldosterone synthesis. They found that Ang II increased aldosterone production by elevating Nox activity and H_2_O_2_ levels in adrenal cortical cells. The elevated H_2_O_2_ then facilitated the activity of aldosterone synthase (CYP11B2), the enzyme responsible for the final steps in aldosterone biosynthesis (5). Elevated reactive oxygen species and reduced radical scavenging capacity have also been linked to adrenal tumor types, and increased ROS are known to promote tumor progression (6, 7).

The NADPH oxidase (Nox) family consists of major ROS-producing enzymes. NADPH oxidase 4 (Nox4) is one of the seven isoforms, and unlike other Nox enzymes, Nox4 is constitutively active and predominantly found in the kidneys, heart, vasculature, and adipose tissue (8). Additionally, Nox4 differs from other NADPH oxidases due to its release of hydrogen peroxide (H_2_O_2_) rather than superoxide anion (9). This characteristic of Nox4 confers a unique role in cellular signaling and homeostasis, as H_2_O_2_ serves as a signaling molecule involved in various processes. In the cardiovascular system, the role of Nox4 has long been described, and its importance has been emphasized (10–14). Recently, we published a study showing the importance of Nox4 in resistant arteries for endothelial function under hypercholesterolemia conditions (15). We also reported that mice with Nox4 knockout become less active, with reduced mitochondrial mass and citrate synthase activity (16). The latest studies have indicated Nox4’s involvement in ferroptosis via mitochondrial dysfunction and lipid peroxidation and also in cardiovascular diseases (17, 18). Nox4 has also been described to drive antioxidant defense response in non-alcoholic fatty liver disease (19). Additionally, NADPH oxidases have been proposed as targets in different diseases such as diabetes, pulmonary fibrosis, and biliary cholangitis (20).

However, the role of Nox4 in adrenal function is not well understood. While ROS generation in various adrenal pathologies has been widely studied, the importance of Nox4 as a source of ROS and its specific role in adrenal gland function under physiological conditions remains unclear. We propose that Nox4-derived H_2_O_2_ may act as a paracrine redox signal within the adrenal microenvironment, potentially influencing both cortical and medullary functions. Hence, we hypothesized that Nox4-produced H_2_O_2_ plays a role in adrenal and catecholamine functions under healthy conditions. In agreement with this hypothesis, we show that hydrogen peroxide induced catecholamine synthesis and release. Vascular alpha-1 adrenergic receptor expression was reduced alongside impaired adrenergic contraction in Nox4-depleted mice, contributing to lower blood pressure. In Nox4^−/−^ mice challenged with voluntary running devices, we detected smaller adrenal size and decreased urine norepinephrine concentrations, directly linking the known decreased physical activity performance in Nox4^−/−^ mice with their adrenal function.

## 2 Materials and methods

### 2.1 Mouse models

All experiments were conducted in compliance with the Guide for the Care and Use of Laboratory Animals published by the US National Institutes of Health (NIH Publication No. 85-23, revised 1996). The animal research ethics committee of TU Dresden and the Regional Council (*Landesdirektion*) Dresden approved both the animal facilities and the experimental procedures in accordance with institutional guidelines and German animal welfare regulations (AZ: DD25-5131/554/11, AZ: DD24.1-5131/450/9, AZ: DD24.1-5131/449/20, and AZ: DD24.1-5131/449/21).

C57BL/6J/Nox4^−/−^ (Nox4^−/−^) and C57BL/6J/Nox4^flox/flox^ mice were generously provided by Ralf P. Brandes and Katrin Schröder (Goethe University, Frankfurt, Germany) and bred in-house. Endothelial cell-specific Nox4^−/−^ mice (EC-Nox4^−/−^) were generated by crossing C57BL/6J/Nox4^flox/flox^ with C57BL/6J/Flk1-Cre^0/+^ mice, followed by continuous genotyping.

C57BL/6J/Ldlr^−/−^ (Ldlr^−/−^) mice were purchased from The Jackson Laboratory via Charles River (Sulzfeld, Germany). Nox4^−/−^ mice were crossed with Ldlr^−/−^ mice in-house for at least five generations and underwent continuous genotyping to generate Nox4^−/−^/Ldlr^−/−^ mice.

### 2.2 Murine tissue sampling

Animals were sacrificed via cervical dislocation; their heart, aorta, and adrenal glands were excised and rinsed with phosphate-buffered saline (PBS), snap-frozen in liquid nitrogen, and stored at −80 °C until further processing.

### 2.3 Animal exercise protocol

At 6 weeks of age, mice were housed individually in cages equipped with a running wheel (Tecniplast, Varese, Italy) for voluntary exercise, as described previously (16). Each complete wheel rotation was recorded, and MATLAB (MathWorks, Natick, USA) was used to calculate individual running distance, daily activity, and velocity. Animals were consistently kept in separate running wheel cages for the same duration.

### 2.4 Echocardiography

A detailed description is provided elsewhere (21). Mice were anaesthetized using isoflurane (1%–1.5%) and placed on a controlled warming pad to maintain a constant body temperature. Transthoracic echocardiography was performed using the Vevo 3100 system and a 15–45-MHz transducer (FUJIFILM VisualSonics Inc., Amsterdam, Netherlands) to assess cardiac structure and function. For systolic function, the B-mode and M-mode of parasternal long and short axes at the papillary muscle level were measured. Diastolic function was assessed in the apical four-chamber view using pulsed-wave Doppler [for measurement of early (E) and atrial (A) waves of the mitral valve velocity] and tissue Doppler (for measurement of myocardial velocity e) at the level of the basal septal segment in the septal wall of the left ventricle. Functional parameters [e.g., left ventricular ejection fraction (LVEF), stroke volume, and E/é] were obtained using the Vevo LAB 5.10.0 software.

### 2.5 Blood pressure measurement

The blood pressure of Ldlr^−/−^ and Nox4^−/−^/Ldlr^−/−^ mice was measured using a non-invasive blood pressure (NIBP) device from ADInstruments (IN125NIBP Controller, MLT125/m Pulse Transducer/Pressure Cuff for NIBP, Australia) using the tail-cuff method. Blood pressure measurements were conducted in a designated quiet area with a regulated temperature (22 °C ± 2 °C) where the mice were kept and allowed to acclimatize for 30 min before the experiment began. Each mouse was guided to walk into the restrainer, and the end holder was adjusted to prevent excessive movement. The tail cuff was placed on the proximal end of the mouse tail, with the pulse transducer located on the ventral surface of the tail, directly below the caudal artery. This was performed under the heating chamber setup. The heating allows an increase in blood flow to the tail, and this is displayed as increased signal amplitude in LabChart. The tail cuff was inflated and deflated for approximately 3–4 cycles to allow the mice to acclimatize to the inflation and deflation process. Blood pressure was taken across three to four consecutive measurements with approximately 1-min resting intervals in the conscious mice. Starting 2 weeks before the measurements, the mice were trained on the blood pressure measurement procedure.

### 2.5 Urine collection

Urine was collected following the 20-week physical activity period. Collection took place between 9:00 am and 10:00 am. Trained and well-handled mice were held over a cold plate to induce spontaneous urination, and the urine was subsequently collected. The samples were centrifuged at 14,000 × *g* for 10 min at 4°C to remove sediments, and the supernatant was retained. Urine samples were stored at −80°C until analysis for creatinine and catecholamines.

### 2.6 Creatinine assay

The creatinine levels in urine samples were measured using a colorimetric assay kit (Cayman Chemical Company, Tallinn, Estonia), following the manufacturer’s instructions. The assay is based on the Jaffe reaction, in which creatinine reacts with alkaline picrate to produce a yellow-orange color that is subsequently diminished under acidic conditions. The difference in absorbance at 500 nm, measured before and after acidification, is proportional to the creatinine concentration. A standard curve was used to determine the creatinine concentration in the samples.

### 2.7 Histology of adrenal glands

For paraffin-embedded tissue sections, freshly isolated adrenal glands were washed with PBS and fixed overnight at 4°C in 4% paraformaldehyde (PFA) in PBS. The tissues were dehydrated through a graded ethanol series and cleared using xylol. Afterward, they were embedded in paraffin, sectioned into 3–5-µm-thick slices using a microtome (Leica Microsystems, Wetzlar, Germany), mounted on glass slides, and stored at room temperature until use. Before staining, the slides were deparaffinized in xylol and rehydrated in a descending ethanol series followed by dH_2_O. Paraffin-fixed sections were subsequently stained using Mayer’s hematoxylin for 10 min, rinsed in dH_2_O for another 10 min, stained with eosin for 1 min, rinsed in dH_2_O, and dehydrated in an ascending ethanol series. Finally, sections were mounted using a xylol-based mounting medium (Merck, Germany). Sections were visualized using a computerized microscope (Zeiss Axiovert 25, Zeiss, Jena, Germany), and staining analysis was conducted using the Fiji imaging software by evaluators who were blinded to the mouse groups.

### 2.8 Detection of extracellular H_2_O_2_ using amplex red assay

Freshly dissected adrenal glands were divided into two pieces and incubated in Krebs–Henseleit buffer on 96-well plates, with or without catalase (380 U/mL). H_2_O_2_-mediated oxidation of Amplex Red, resulting in red fluorescence, was measured using a microplate reader (FLUOstar, BMG Labtech, Ortenberg, Germany) with fluorescence excitation at 544 nm and emission at 590 nm under light-protected conditions. H_2_O_2_ production was quantified as the catalase-sensitive part and normalized to tissue weight.

### 2.9 Cell culture

The rat pheochromocytoma cell line PC12 was kindly provided by Arthur Tischler (Department of Pathology and Laboratory Medicine, Tufts University School of Medicine, Boston, MA, USA) and cultivated as previously described (22).

For the catecholamine secretion assay, PC12 cells (10^5^ cells/well) were seeded in collagen A-coated 24-well plates. After 24 h, cells were treated with or without 1 µM dexamethasone for 2 days. Subsequently, cells were treated with either 10 µM or 50 µM H_2_O_2_ (4 h), 2 mM CaCl_2_ (15 min), or Locke’s buffer alone (154 mM NaCl, 5.6 mM KCl, 3.6 mM NaHCO_3_, 5 mM Hepes, and 5.6 mM glucose, pH 7.3). Supernatants were collected, centrifuged, and stored at −80°C.

### 2.10 Catecholamine measurements

Catecholamine levels in mouse adrenal glands and cell culture supernatants were analyzed via high-performance liquid chromatography with electrochemical detection (HPLC–ECD) as previously described (23). Catecholamine levels in mouse urine samples were analyzed via liquid chromatography tandem mass spectrometry (LC-MS/MS) as described elsewhere (24). Concentrations of catecholamines were calculated relative to creatinine concentrations in the urine.

### 2.11 Real-time PCR

Tissue samples were homogenized using the Precellys homogenizer (VWR, Peqlab, Erlangen, Germany), and total RNA was subsequently isolated using peqGOLD (VWR, Peqlab, Erlangen, Germany). For treated cells, RNA extraction was performed using the High Pure RNA Isolation Kit (Roche Diagnostics, Mannheim, Germany). Reverse transcription was conducted using SuperScript II Reverse Transcriptase according to the manufacturer’s instructions (Thermo Fisher Scientific, Waltham, MA, USA), employing 500 ng of total RNA and random hexamer primers. Quantification analysis was performed through real-time PCR using the GoTaq qPCR Master Mix (Promega, Mannheim, Germany). Raw data were analyzed using 7500 software version 2.06 (Applied Biosystems by Life Technologies, Darmstadt, Germany), and results were evaluated using a mathematical model for relative expression ratios in real-time PCR with constant reference gene expression (25). The primers used are detailed in **Table 1**, with ribosomal protein L32 (Rpl32) serving as the reference gene.

**Table 1:** Primers used for real-time PCR.

Gene	Primers	Sequence, 5'-3'	Accession number
*Adra1a* murine	Forward	TGCTAAGGACTGGGTCTTGG	NM_001271759.2
	Reverse	GCCCTGGAGCTTCGTTTATCT	
*Adra1b* murine	Forward	GGATAGCAGACATCATTGGGC	NM_001284380.1
	Reverse	GGCTCGCTCGCCTCT	
*Adra1d* murine	Forward	CTCCGTAAGGCTGCTCAAGT	NM_013460.5
	Reverse	GACGCCCTCTGATGGTTTCA	
*Adra2a* murine	Forward	AGCTGCAAGATCAACGACCA	NM_007417.5
	Reverse	ACGCTTGGCGATCTGGTAAA	
*Adra2b* murine	Forward	GAAAACTTCTCTGGGGCGGA	NM_009633.4
	Reverse	CACCCAGGACCATCTATCGC	
*Adra2c* murine	Forward	AGGACTTCAGGCGCTCTTTC	NM_007418.3
	Reverse	AGAGGGTCATTGCCTGAAGC	
*AdrB1* murine	Forward	ACTGTGGACAGCGATTCGAG	NM_007419.3
	Reverse	GCCTGGCTCTCTACACCTTG	
*AdrB2 murine*	Forward	TGCCTAGCCTTAGCGTTGAC	NM_007420.3
	Reverse	TCCTGTCAAGGAGGGGTCTT	
*Dbh* murine	Forward	GTCTACCCGGGGGACG	NM_138942.3
	Reverse	AGAGTTCCAGCTCTGTCTGG	
*Dbh* rat	Forward	ACTCAACTACTGTCGCCACG	NM_013158.2
	Reverse	GCGCCGGCCTTGTATATTCC	
*Nox4* murine	Forward	TGTTGGGCCTAGGATTGTGTT	NM_001285833.1; NM_001285835.1; NM_015760.5
	Reverse	AGGGACCTTCTGTGATCCTCG	
*Nox4* rat	Forward	CCAGATGTTGGGCCTAGGATT	NM_053524.1
	Reverse	GTTCACCAAGTGGGCAGCTA	
*Pnmt* murine	Forward	CGCCTATCTCCGCAACAACT	NM_008890.3
	Reverse	GACACCTCACCGGTAGCAAAG	
*Ryr3* rat	Forward	AATGACTCTCAGCACAGGAGGG	XM_017592338.1, XM_017601129.1
	Reverse	GAAGGCTGTGGACTTGGTGTC	
*Rpl32* murine	Forward	CTGGCCCTTGAACCTTCTCCGC	NM_172086.2
	Reverse	GCGCTGCCTACGAGGTGGCTG	
*Rpl32* rat	Forward	GGTGAAGCCCAAGATCGTCAA	NM_013226.2
	Reverse	TCTGGGTTTCCGCCAGTTTC	

### 2.12 *Ex vivo* analysis of vascular function

Vascular function was analyzed using the DMT Multi Wire Myograph System–Model 620 M (Danish Myo Technology, Hinnerup, Denmark). Aortic segments were isolated and mounted on pins for isometric tension recordings. The mesenteric arteries were mounted on wires for recordings. The organ bath was filled with the Krebs–Henseleit buffer (4.7 mM KCl, 118 mM NaCl, 1.17 mM MgSO_4_, 1.18 mM KH_2_PO_4_, 25 mM NaHCO_3_, 10 mM glucose, 0.026 mM EDTA, and 1.6 mM CaCl_2_) aerated with carbogen 5% (v/v) CO_2_ in O_2_. The vessels were equilibrated at 37°C before contraction was initiated using 80 mM high-potassium Krebs–Henseleit buffer. After multiple washes, phenylephrine (PE)-induced contraction was evaluated by applying increasing concentrations of PE (0.001–30 µmol/L). Concentration–response curves for U4611 were measured by applying increasing concentrations of U46619 (0.001–3 µmol/L) to the organ bath.

### 2.13 Statistical analyses

Data are presented as mean ± standard deviation (SD). Normal (Gaussian) distribution was tested using the Shapiro–Wilk normality test. Non-Gaussian distributed data were analyzed using the Kruskal–Wallis and Dunn’s multiple comparison tests. Gaussian-distributed data were analyzed with t-tests or one-way ANOVAs followed by Tukey’s multiple comparisons test (GraphPad Prism 9, GraphPad Software, Inc., La Jolla, USA). *p* < 0.05 was considered statistically significant.

## 3 Results

### 3.1 NADPH oxidase 4 expression in the adrenal gland

Nox4 is expressed in the adrenal glands of wild-type (WT) mice. Additionally, Nox4 mRNA expression increased with age: older mice (26-week-old) showed 1.8-fold higher expression compared with younger mice (10-week-old) (**Figure 1A**). As an indicator of Nox4 activity, we measured catalase-sensitive hydrogen peroxide release from the adrenal glands. Significantly reduced hydrogen peroxide concentrations were detectable in the adrenal glands of Nox4^−/−^ mice (**Figure 1B**).

**Figure 1:**
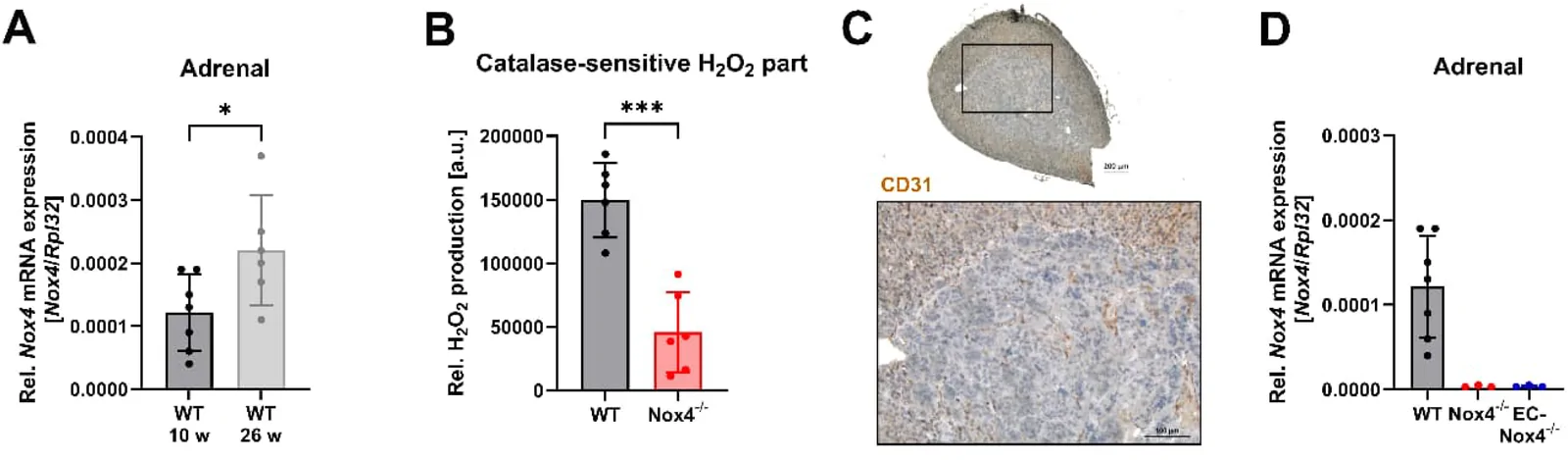
**(A)** Nox4 mRNA expression in adrenal glands of 10-week-old (10 w) and 26-week-old (26 w) WT mice (n ≥ 6). **(B)** Catalase-sensitive H_2_O_2_ release from adrenal glands of 10-week-old WT and Nox4^−/−^ mice (n = 6). **(C)** Endothelial cells in the adrenal gland. Representative image of CD31 staining in the left murine adrenal gland. **(D)** Nox4 mRNA expression in adrenal glands of 10-week-old WT, Nox4^−/−^, and endothelial cell-specific Nox4^−/−^ mice (n = 6). Statistics: Data were analyzed with t-tests or one-way ANOVAs followed by Tukey’s multiple comparisons test. *p < 0.05; **p < 0.01; ***p < 0.001. EC-Nox4^−/−^, endothelial cell-specific NADPH oxidase 4 knockout; Nox4, NADPH oxidase 4; Rpl32, ribosomal protein L32; WT, wild type.

The highest expression of Nox4 is found in endothelial cells. Therefore, adrenal glands were stained for the endothelial cell marker CD31. This revealed that endothelial cells were spread throughout the entire adrenal gland (**Figure 1C**). The adrenal glands of generated endothelial cell-specific Nox4-knockout mice (EC-Nox4^−/−^) revealed no detectable Nox4 expression, similar to global Nox4^−/−^ mice (**Figure 1D**). We therefore proposed that intratissue crosstalk occurs between H_2_O_2_-source Nox4 in the endothelial cells and surrounding adrenal tissue cells.

### 3.2 Deletion of Nox4 resulted in decreased catecholamine levels in the adrenal gland

Analysis of the catecholamine profile in the adrenal glands of WT and global Nox4^−/−^ mice revealed no differences in tissue dopamine levels (**Figure 2A**), but a decrease in norepinephrine concentrations in Nox4-depleted mice (**Figure 2C**). Similarly, we detected reduced tissue concentrations of epinephrine in the adrenal glands of Nox4^−/−^ mice compared with the adrenal glands of WT mice (**Figure 2E**). The mRNA expression of enzymes in the catecholamine pathway showed decreased dopamine beta-hydroxylase expression in Nox4^−/−^ mice, indicating a first explanation for the decreased norepinephrine concentrations in Nox4^−/−^ adrenal glands (**Figure 2B**). Although we observed upregulated phenylethanolamine *N*-methyltransferase mRNA expression, this appeared not to be accompanied by increased enzymatic activity (**Figure 2D**), as indicated by the decreased epinephrine levels in the adrenal glands of Nox4^−/−^ mice.

**Figure 2:**
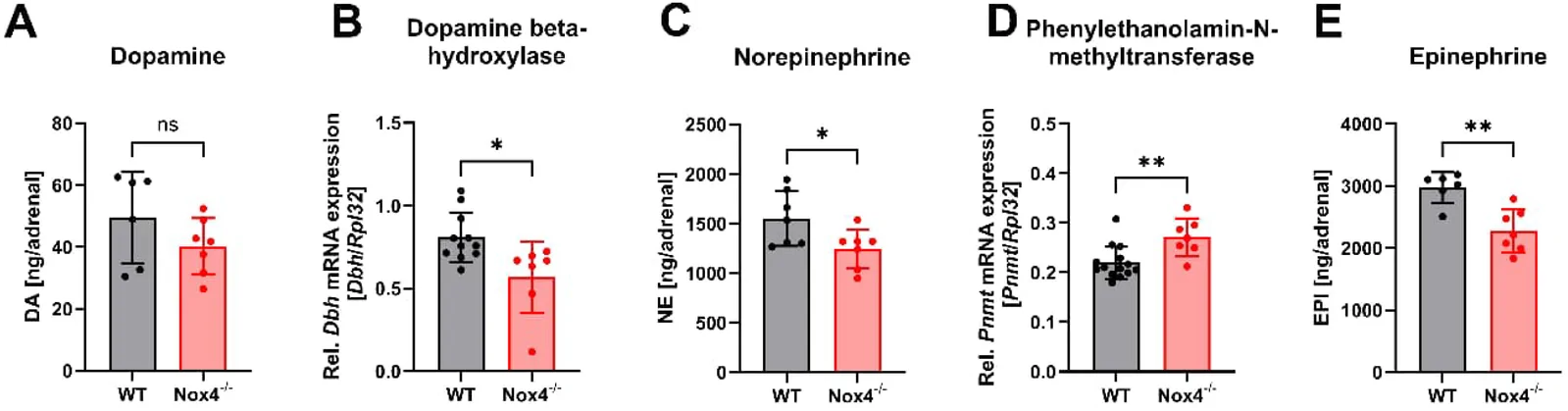
**(A)** Dopamine expression in adrenal glands of 10-week-old WT and total Nox4^−/−^ mice (n = 7). **(B)** Dopamine beta-hydroxylase mRNA expression in adrenal glands of WT and total Nox4^−/−^ mice (n ≥ 7). **(C)** Norepinephrine expression in adrenal glands of WT and total Nox4^−/−^ mice (n = 7). **(D)** Phenylethanolamine *N*-methyltransferase mRNA expression in adrenal glands of WT and total Nox4^−/−^ mice (n = 7). **(E)** Epinephrine expression in adrenal glands of WT and total Nox4^−/−^ mice (n = 7). Statistics: t-tests; *p < 0.05; **p < 0.01. DA, dopamine; Dbh, dopamine beta-hydroxylase; EPI, epinephrine; NE, norepinephrine; Nox4^−/−^, NADPH oxidase 4 knockout; Pnmt, phenylethanolamine *N*-methyltransferase; Rpl32, ribosomal protein L32; WT, wild type.

### 3.3 Hydrogen peroxide-induced catecholamine secretion and DBH expression in murine chromaffin cells

To model chromaffin cells *in vitro*, we employed the rat pheochromocytoma-derived PC12 cell line, which does not express Nox4 (**Figure 3A**). Using this model, we studied norepinephrine synthesis and release following external stimulation, such as hydrogen peroxide (**Figure 3B**). For control experiments, we used dexamethasone treatment, which is known to stimulate catecholamine synthesis (26). After the application of 1 µM dexamethasone, we were able to detect 2.8-fold increased dihydroxyphenylalanine (DOPA) (**Figure 3C**), 3.5-fold increased dopamine (**Figure 3D**), and 2-fold increased norepinephrine concentrations (**Figure 3E**) in the supernatant of PC12 cells. Furthermore, it has been shown that increasing extracellular Ca^2+^ concentrations promotes catecholamine release from neuroendocrine cells, triggering exocytosis of catecholamine-containing vesicles (27). In our model, we confirmed that catecholamine secretion can be induced by adding 2 mM CaCl_2_. We observed 1.7-fold higher DOPA (**Figure 3F**), 3.5-fold higher dopamine (**Figure 3G**), and 2.6-fold higher norepinephrine concentrations (**Figure 3H**) in the supernatant of PC12 cells treated with CaCl_2_.

**Figure 3:**
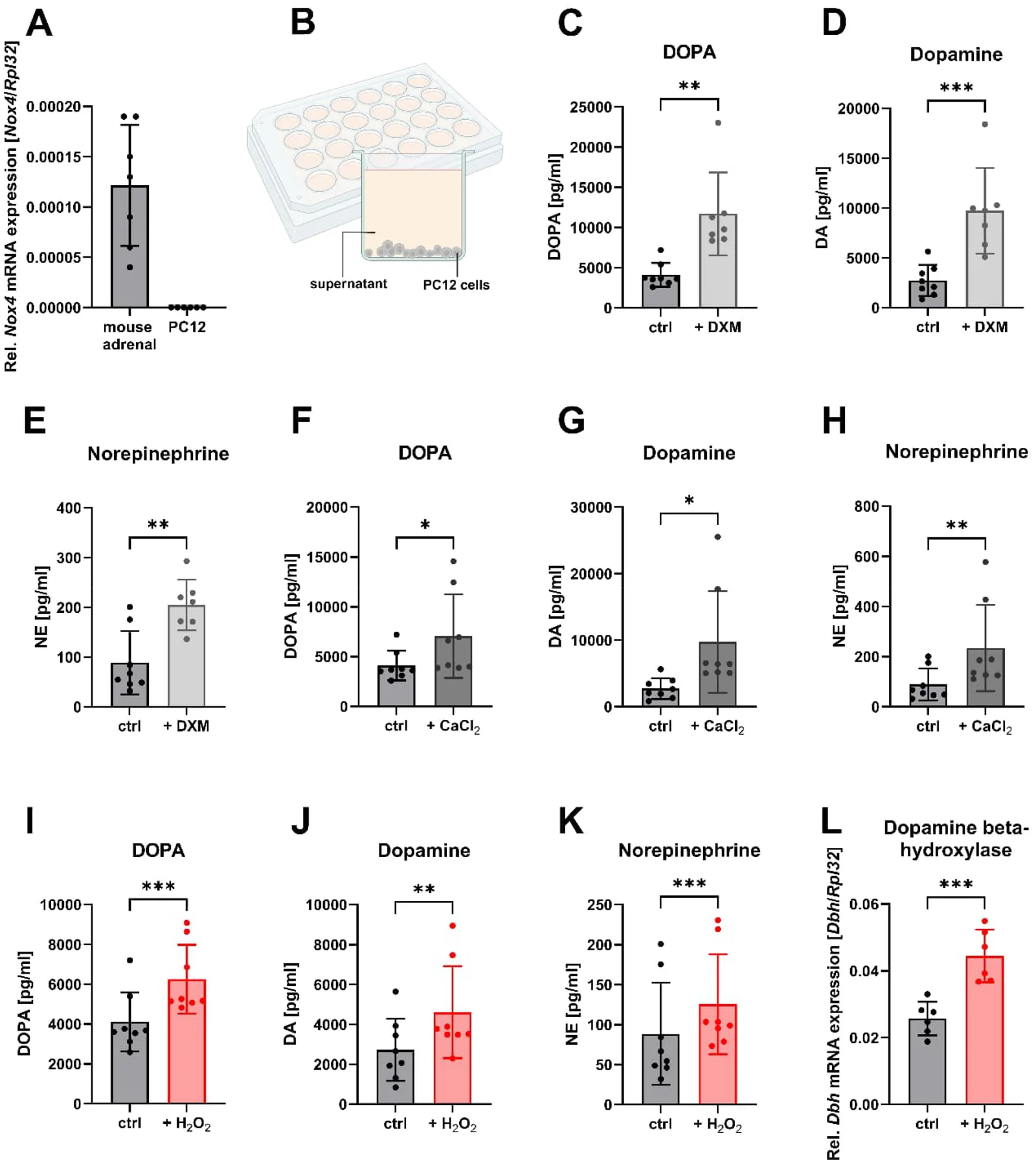
Hydrogen peroxide led to increased catecholamine secretion in the supernatant of chromaffin cells. **(A)** Nox4 mRNA expression in murine adrenal and PC12 cells (n ≥ 6). **(B)** Graphical illustration of experimental setup for PC12 cells and analyzed supernatant. Graphic design was created with BioRender. **(C)** DOPA concentration, **(D)** dopamine concentration, and **(E)** norepinephrine concentration in supernatant of control PC12 cells and PC12 cells treated with 1 µM dexamethasone (+DXM) (n ≥ 7). **(F)** DOPA concentration, **(G)** dopamine concentration, and **(H)** norepinephrine concentration in supernatant of control PC12 cells and cells treated with 2 mM CaCl_2_ (+CaCl_2_) (n = 8). **(I)** DOPA concentration, **(J)** dopamine concentration, and **(K)** norepinephrine concentration in supernatant of control PC12 cells and cells treated with 10 µM H_2_O_2_ (+H_2_O_2_) (n = 8). **(L)** Dopamine beta-hydroxylase mRNA expression in control PC12 cells and cells treated with 100 µM H_2_O_2_ (+H_2_O_2_) (n = 6). Statistics: t-test; *p < 0.05; **p < 0.01; ***p < 0.001. DA, dopamine; Dbh, dopamine beta-hydroxylase; DOPA, dihydroxyphenylalanine; NE, norepinephrine; Nox4, NADPH oxidase 4; Rpl32, ribosomal protein L32.

To assess whether endothelial Nox4-derived H_2_O_2_ may influence catecholamine secretion from chromaffin cells, we applied exogenous H_2_O_2._ Treatment of PC12 cells with 10 µM hydrogen peroxide resulted in 1.5-fold elevated DOPA (**Figure 3I**), 1.6-fold elevated dopamine (**Figure 3J**), and 1.4-fold elevated norepinephrine concentrations (**Figure 3K**) in the supernatant. In addition, we detected upregulated dopamine beta-hydroxylase expression in PC12 cells treated with hydrogen peroxide (**Figure 3L**), which could be evidence of endothelial Nox4-derived H_2_O_2_ being able to affect catecholamine synthesis and secretion.

### 3.4 Differential catecholamine profile in Nox4-depleted mice reduced adrenergic contraction

One of the most prominent physiological targets of catecholamines is the cardiovascular system, particularly adrenergic receptors on blood vessels. The analysis of the vasculature revealed the downregulation of norepinephrine and epinephrine alpha-1A adrenergic receptor (Adra1a) (**Figure 4A**) and alpha-1D adrenergic receptor (Adra1d) (**Figure 4B**) in the aorta of Nox4^−/−^ mice. For functional analysis, we performed PE-induced contraction measurements. Concentration–response curves revealed a significant reduction in PE contraction in aortic rings of Nox4^−/−^ mice (**Figure 4C**). By contrast, contraction induced by the thromboxane A_2_ analogue U46619 was comparable between WT and Nox4^−/−^ mice (**Figure 4D**). Likewise, receptor-independent contraction induced by 80 mM KCl did not differ between genotypes (**Figure 4E**). Smooth muscle relaxation, assessed by cumulative administration of sodium nitroprusside (SNP), was also comparable between WT and Nox4^−/−^ mice, indicating preserved smooth muscle relaxation capacity (**Figure 4F**). Overall, these findings suggest that Nox4 deficiency induces pathway-specific alterations in adrenergic vascular reactivity, while global smooth muscle function remains preserved.

**Figure 4:**
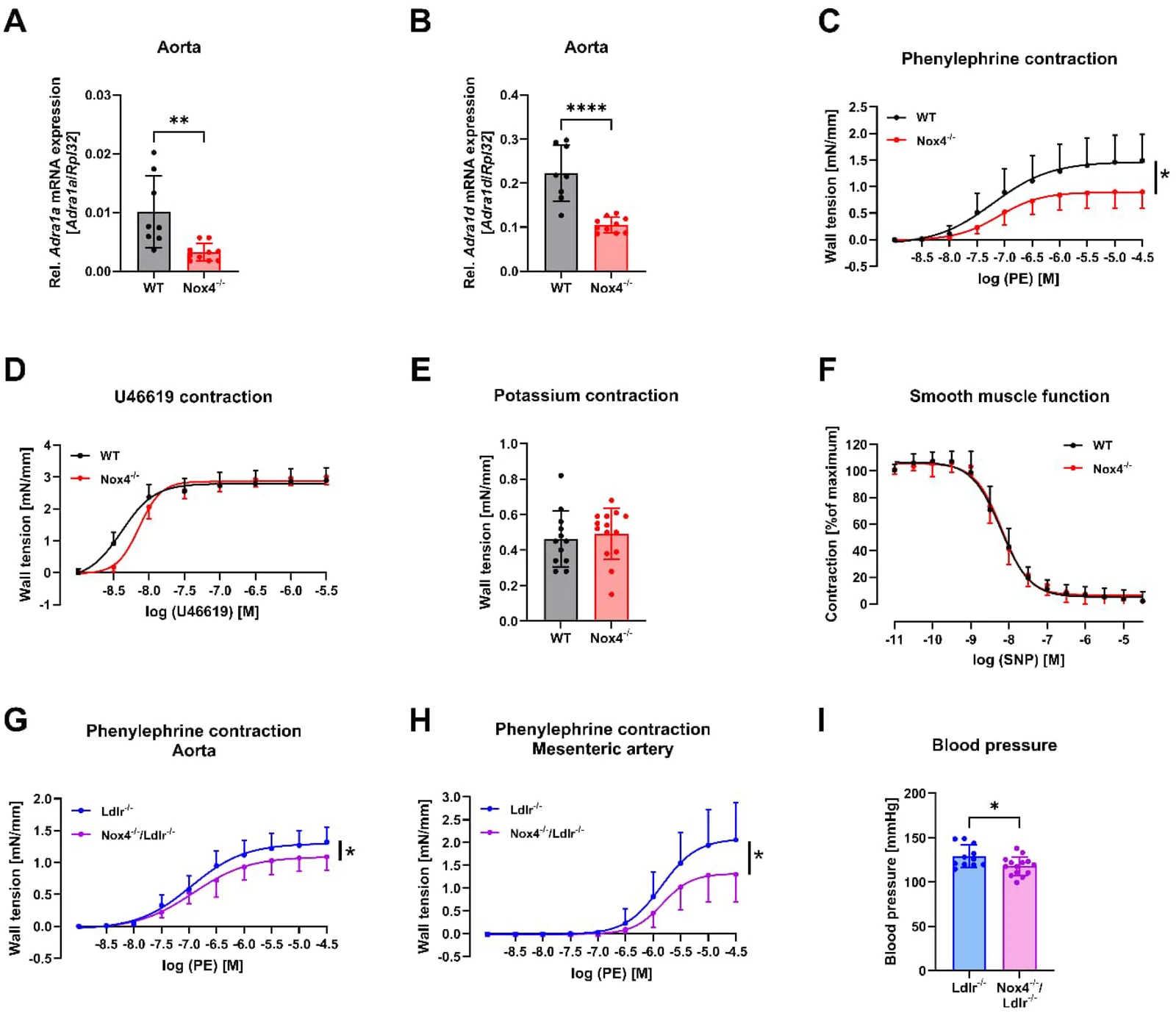
Reduction in alpha-1 adrenergic receptor mRNA expression in Nox4^−/−^ mice decreased PE-induced vascular contraction. **(A)** Alpha-1A adrenergic receptor (Adra1a) mRNA expression in aortas from WT and Nox4^−/−^ mice (n ≥ 8). **(B)** Alpha-1D adrenergic receptor (Adra1d) mRNA expression in aortas from WT and Nox4^−/−^ mice (n ≥ 8). **(C)** Concentration–response curves for phenylephrine in aortic segments from 10-week-old WT and Nox4^−/−^ mice (n ≥ 12). **(D)** Concentration–response curves for U46619 in aortic segments from WT and Nox4^−/−^ mice (n ≥ 8). **(E)** Contraction induced by 80 mM KCl in arteries from WT and Nox4^−/−^ mice (n ≥ 12). **(F)** Concentration–response curves for sodium nitroprusside in aortic segments from WT and Nox4^−/−^ mice (n ≥ 8). **(G)** Concentration–response curves for phenylephrine in aortic segments from Ldlr^−/−^ and Nox4^−/−^/Ldlr^−/−^ mice (n ≥ 8). **(H)** Concentration–response curves for phenylephrine in mesenteric arteries from Ldlr^−/−^ and Nox4^−/−^/Ldlr^−/−^ mice (n ≥ 8). **(I)** Systolic mean blood pressure in Ldlr^−/−^ and Nox4^−/−^/Ldlr^−/−^ mice (n ≥ 11). Statistics: t-test; *p < 0.05; **p < 0.01; ***p < 0.001; ****p < 0.0001. Adra1a, alpha-1A adrenergic receptor; Adra1d, alpha-1D adrenergic receptor; Ldlr^−/−^, low-density lipoprotein receptor knockout; Nox4^−/−^, NADPH oxidase 4 knockout; PE, phenylephrine; Rpl32, ribosomal protein L32; SNP, sodium nitroprusside; WT, wild type.

In a more pathological setting of atherosclerosis-prone Ldlr^−/−^ mice, Nox4 knockout was also associated with reduced PE-induced contraction in aortic rings (**Figure 4G**), an effect that was even more pronounced in the mesenteric arteries (**Figure 4H**). Assessing blood pressure non-invasively revealed lower mean systolic blood pressure in Nox4^−/−^/Ldlr^−/−^ mice (**Figure 4I**).

The lower concentrations of norepinephrine and epinephrine in Nox4^−/−^ mice, along with the reduced alpha-1A adrenergic receptor expression, likely resulted in the observed diminished phenylephrine-induced vasoconstriction. To investigate their potential effects on the heart, the mice were examined via echocardiography.

### 3.5 Cardiac function was unaffected in Nox4^−/−^ mice

The 10-week-old mice were examined via echocardiography (**Figure 5E**), and heart tissue was obtained. We observed similar mRNA expression of alpha-1A adrenergic receptor (Adra1a), alpha-1D 365 adrenergic receptor (Adra1d), and beta-2 adrenergic receptor between WT and Nox4^−/−^ mice, whereas beta-1 adrenergic receptor mRNA expression was upregulated in the heart tissue from knockout mice (**Figures 5A–D**). This may represent a compensatory response to reduced circulating catecholamines, as the heart tried to maintain adrenergic sensitivity despite lower norepinephrine and epinephrine levels in the Nox4^−/−^ mice.

**Figure 5:**
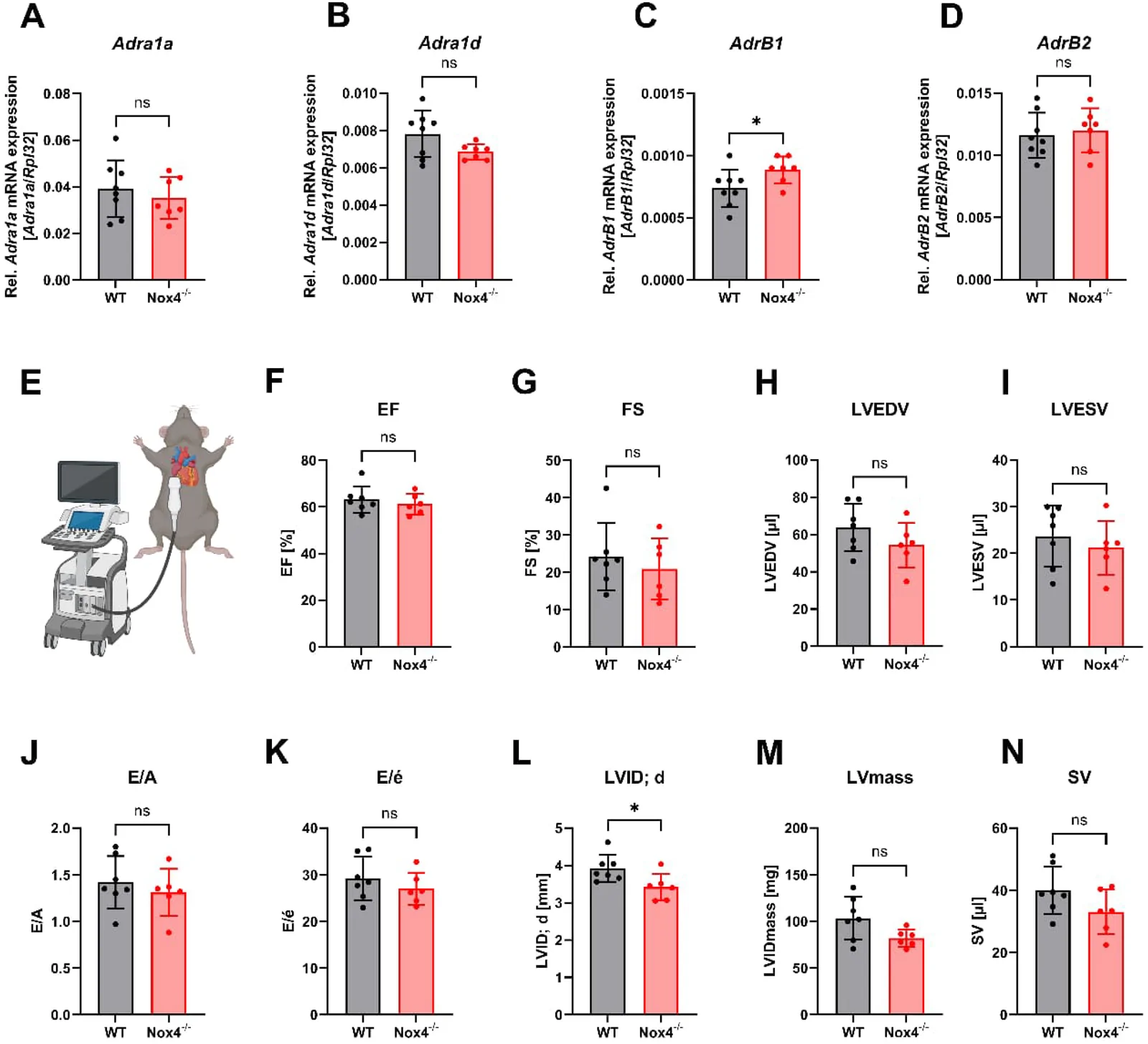
Similar myocardial performance in WT and Nox4^−/−^ mice. **(A)** Alpha-1A adrenergic receptor (Adra1a) mRNA expression, **(B)** alpha-1D adrenergic receptor (Adra1d), **(C)** beta-1 adrenergic receptor (AdrB1) mRNA expression, and **(D)** beta-2 adrenergic receptor (AdrB2) mRNA expression in hearts of 10-week-old WT and Nox4^−/−^ mice (n ≥ 7). **(E)** Echocardiography in 10-week-old mice. Graphic design was created with BioRender. **(F)** Left ventricular ejection fraction (LVEF), **(G)** fractional shortening (LVFS), **(H)** left ventricular end-diastolic volume (LVEDV), **(I)** left ventricular end-systolic volume (LVESV), **(J)** ratio of early mitral inflow to late mitral inflow (E/A), **(K)** ratio of early mitral inflow to tissue Doppler mitral annulus velocity in early diastole (E/é), **(L)** end-diastolic left ventricular internal diameter (LVID; d), **(M)** left ventricular mass (LVmass), and **(N)** stroke volume (SV) in hearts of 10-week-old WT and Nox4^−/−^ mice (n = 7). Statistics: t-test; *p < 0.05. A, late mitral inflow; Adra1a, alpha-1A adrenergic receptor; Adra1d, alpha-1D adrenergic receptor; AdrB1, beta-1 adrenergic receptor; AdrB2, beta-2 adrenergic receptor; E, early mitral inflow; é, tissue Doppler mitral annulus velocity in early diastole; LVEDV, left ventricular end-diastolic volume; LVEF, left ventricular ejection fraction; LVESV, left ventricular end-systolic volume; LVFS, left ventricular fractional shortening; LVID, left ventricular internal diameter; Nox4, NADPH oxidase 4; Rpl32, ribosomal protein L32; SV, stroke volume; WT, wild type.

The overall myocardial performance (**Figures 5F–N**) was similar between WT and Nox4^−/−^ mice. However, in the end-diastolic left ventricular internal diameter (LVID), we observed a significant reduction in Nox4^−/−^ mice (**Figure 5L**).

Lower norepinephrine and epinephrine levels could reduce sympathetic stimulation of the heart, leading to a decrease in ventricular filling pressures and preload. Despite the smaller LVID, unchanged stroke volume (**Figure 5N**) and ejection fraction (**Figure 5F**) suggest that the heart compensates effectively, potentially through improved myocardial efficiency or compliance. The reduction in LVID may indicate a form of adaptive remodeling, where the heart adjusts to lower catecholamine levels. This could happen without compromising pump function. These observations suggest that the cardiac system maintains functional stability despite the changes in neurohormonal signaling.

### 3.6 Depletion of Nox4 affected adrenal phenotype and urine norepinephrine concentrations of physically active mice

Physical exercise stimulates the sympathetic nervous system, leading to the release of norepinephrine from the adrenal medulla and nerve endings to prepare the body for increased physical demands (28). Norepinephrine increases heart rate and blood pressure, enhancing blood flow to muscles. It causes vasoconstriction in non-essential areas, redirecting blood to active muscles. WT and Nox4^−/−^ mice were challenged with voluntary running devices for 20 weeks. In previous studies, we and others have shown that Nox4^−/−^ mice become less active and run less compared with WT mice (16, 29). The novelty of the present study was that we aimed to investigate whether the catecholamine profile is affected in Nox4-knockout mice, potentially providing further insights into the previously reported reduced exercise capacity. Therefore, the adrenal glands and urine of each mouse were collected after 20 weeks of voluntary running (**Figure 6A**). Surprisingly, right and left adrenal weights were significantly decreased in Nox4^−/−^ mice compared with WT mice (**Figures 6B, C**). Histological imaging and quantification also revealed reduced adrenal size in Nox4^−/−^ mice (**Figures 6D, E**), characterized by significantly smaller cortical and medullary areas compared with those in controls (**Figures 6F, G**). The analysis of urine samples revealed no differences in dopamine concentrations (**Figure 6H**), but 30% lower norepinephrine levels in Nox4^−/−^ mice (**Figure 6I**). For epinephrine, we only detected a tendency toward reduction in the urine of Nox4^−/−^ mice (**Figure 6J**).

**Figure 6:**
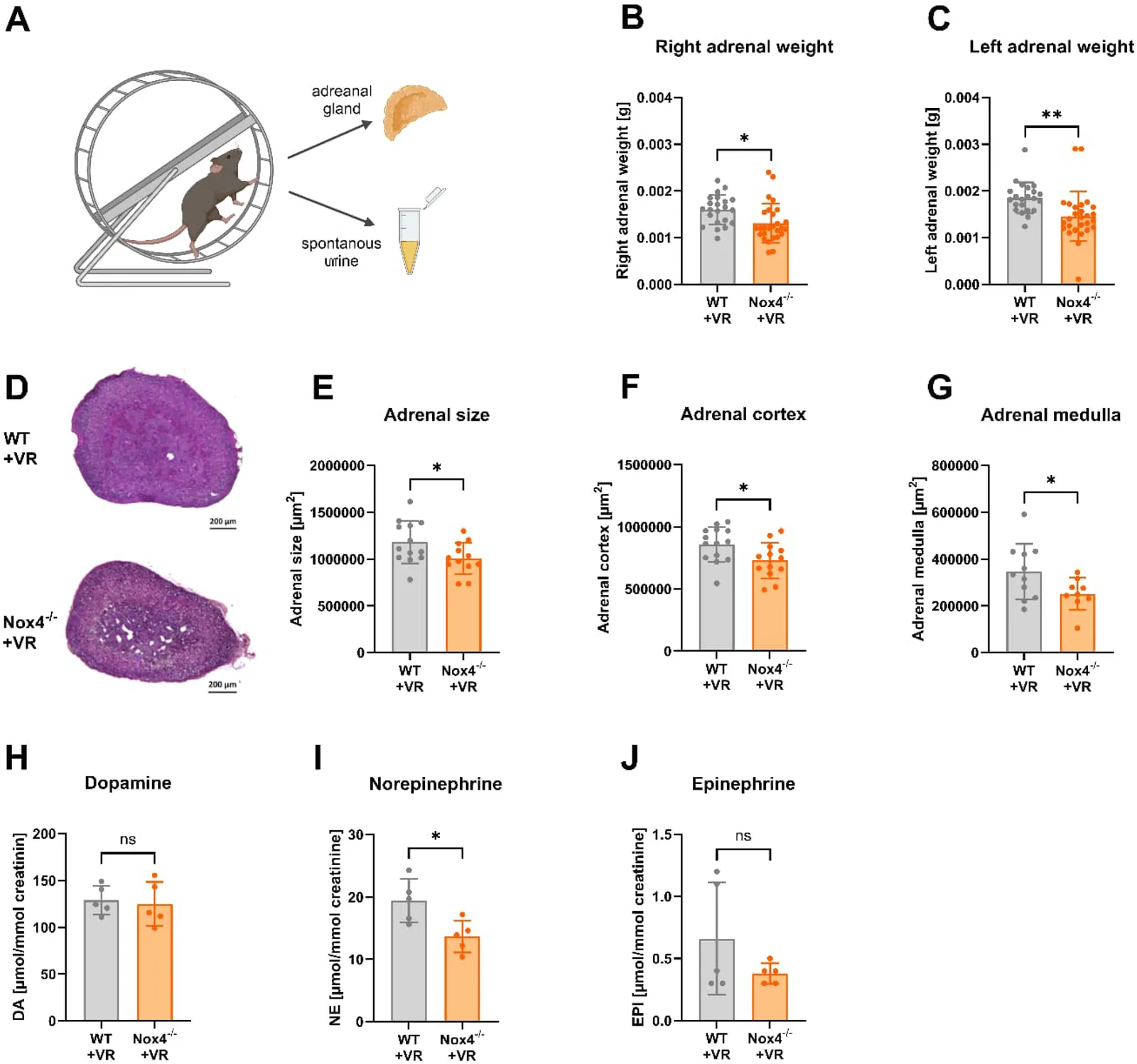
Effects in physically active mice. **(A)** WT and Nox4^−/−^ mice had access to voluntary running wheels for 20 weeks. At 20th week, urine and adrenal glands were collected. Graphic design was created with BioRender. **(B)** Weight of right adrenal gland and **(C)** weight of left adrenal gland from WT and Nox4^−/−^ mice after 20 weeks of voluntary running (n ≥ 22). **(D)** Representative image of H&E staining of left murine adrenal glands of WT and Nox4^−/−^ mice after 20 weeks of voluntary running. **(E)** Quantification of histology images of whole adrenal size from WT and Nox4^−/−^ mice after 20 weeks of voluntary running (n ≥ 13). **(F)** Area of adrenal cortex in histology images from WT and Nox4^−/−^ mice after 20 weeks of voluntary running (n = 13). **(G)** Area of adrenal medulla in histology images from WT and Nox4^−/−^ mice after 20 weeks of voluntary running (n ≥ 13). **(H)** Dopamine concentration, **(I)** norepinephrine concentration, and **(J)** epinephrine concentration relative to creatinine concentrations in the urine of WT and Nox4^−/−^ mice after 20 weeks of voluntary running (n = 5). Statistics: t-test; *p < 0.05; **p < 0.01. DA, dopamine; EPI, epinephrine; H&E, hematoxylin and eosin; NE, norepinephrine; Nox4, NADPH oxidase 4; Rpl32, ribosomal protein L32; VR, voluntary running; WT, wild type.

In addition, we performed echocardiographic examinations on the mice following the 20-week voluntary running period. We did not detect any differences in myocardial performance between active WT and Nox4^−/−^ mice (**Supplementary Figures 1A–J**).

## 4 Discussion

Here, we present evidence for the effects of Nox4 on adrenal medullary function, particularly norepinephrine homeostasis and effects on vascular contraction.

Nox4 was age-dependently expressed in the adrenal gland, and its deletion led to significantly reduced hydrogen peroxide release. The adrenal gland is one of the most highly vascularized organs, with its vascular network playing a crucial role throughout life by supplying precursors for steroidogenesis and facilitating the secretion of hormones into the blood (30). A close interaction between endothelial and adrenal cells persists (31). Consequently, we generated an endothelial-specific knockout (EC-Nox4^−/−^) mouse and could not detect Nox4 expression in the adrenal glands.

The deletion of Nox4 resulted in reduced norepinephrine concentrations with decreased dopamine beta-hydroxylase expression in the adrenal glands of the Nox4^−/−^ mice compared with WT mice. Additionally, reduced epinephrine concentrations were observed in those mice, while phenylethanolamine *N*-methyltransferase mRNA expression was increased in Nox4^−/−^ mice. Therefore, Nox4-released hydrogen peroxide might affect catecholamine concentrations at several levels. It has been shown that reactive oxygen species can modulate the activity of key enzymes of the catecholamine synthesis pathway, particularly tyrosine hydroxylase (32) and phenylethanolamine *N*-methyltransferase (33). In our *in vitro* experiments, we observed that treatment of chromaffin cells with hydrogen peroxide increased dopamine beta-hydroxylase expression and stimulated catecholamine release. An elevated catecholamine synthesis caused by ROS has also been reported in rat adrenal medulla, where hypoxia led to ROS-induced dopamine beta-hydroxylase expression (34). H_2_O_2_ has been shown to activate pathways including MAPK/AP-1 signaling (35), while AP-1 and CRB response elements in the Dbh promoter have been implicated (36). Another transcription factor regulating Dbh expression is Egr1, which has also been described as redox-sensitive (37). Interestingly, it has been reported that Egr1 can activate Nox4 expression in other cellular systems (38).

The release of norepinephrine from norepinephrine storage vesicles within chromaffin cells in response to stimuli might also be affected by Nox4. Stimulation leads to an influx of calcium ions (Ca^2+^), which is crucial for vesicle fusion and exocytosis (39). Souvannakitti et al. observed that Nox4 influenced this process through ROS production, which affected calcium signaling pathways. Nox4 enhanced the recruitment of T-type Ca^2+^ channels and ryanodine receptors, modulating intracellular calcium stores in adrenal chromaffin cells, thus facilitating norepinephrine release (40). Interestingly, Infanger et al. demonstrated that the silencing of Nox4 in the hypothalamic paraventricular nucleus attenuated sympathetic outflow and markedly reduced urinary norepinephrine levels in a mouse model of myocardial infarction (41). Altered Nox4-dependent sympathetic signaling may, therefore, also contribute to our observed results.

Further analysis of the functional consequences of the reduced norepinephrine levels in Nox4^−/−^ mice revealed decreased alpha-1 adrenergic receptor expression and impaired phenylephrine-induced vascular contraction. This diminished adrenergic vasoconstriction might contribute to reduced systemic vascular resistance and lower blood pressure, as we were able to prove in the atherosclerosis-prone Ldlr^−/−^ mouse model with additional depletion of Nox4. Here, we detected significantly lower mean systolic blood pressure. Rezende et al. also showed that a low-sodium diet caused reduced blood pressure in Nox4^−/−^ mice compared with wild-type mice. The authors claimed that this was a consequence of reduced peripheral resistance, rather than altered sodium handling (42). Nanduri et al. reported that inhibiting HIF-1alpha-dependent activation of Nox4 in rats resulted in reduced plasma norepinephrine concentrations and reduced blood pressure (43). Our observed phenotype of reduced blood pressure being caused by the diminished alpha-1 adrenergic receptor expression in the vasculature of the Nox4^−/−^ mice is consistent with the findings that mice lacking the alpha-1 adrenergic receptor showed reduced mean arterial blood pressure and decreased aortic ring contraction in response to phenylephrine (44). This was proven for several alpha-1 adrenergic subtypes. Transgenic mice with alpha-1B adrenergic receptor knockout were protected against chronic increase of blood pressure induced by norepinephrine (45). Moreover, mice lacking the alpha-1A adrenergic receptor subtype displayed, in addition to lower basal blood pressure, an attenuated blood pressure response to phenylephrine and norepinephrine (46). A reduced basal blood pressure and a diminished aortic contraction response to norepinephrine and phenylephrine were also reported for mice deficient in the alpha-1D adrenergic receptor subtype (47). Furthermore, the absence of alpha-1D adrenergic receptors led to reduced salt-induced hypertension (48). Notably, hypoxia is known to increase alpha-adrenergic signaling in human vasculature (49), which might depend on Nox4 that is also upregulated under hypoxic conditions (50).

Consequently, lower norepinephrine and reduced alpha-1 adrenergic receptor expression might protect against conditions associated with excessive sympathetic activity, such as hypertension and vascular remodeling or hypertrophy. In chronic heart failure, high norepinephrine levels and adrenergic overstimulation are detrimental. The analyses in our animal model were performed under physiological conditions and indicated only a slight difference in left ventricular internal diameter between WT and Nox4^−/−^ mice, while other heart parameters were unaffected. However, under pathological conditions, studies have shown that Nox4 is important for cardiac function. Zhang et al. detected contractile dysfunction of the heart of Nox4^−/−^ mice in a model of pressure overload due to aortic banding (13). Likewise, specific deletion of Nox4 in cardiomyocytes or endothelial cells led to pressure overload-induced cardiac dysfunction (51, 52). Nox4 expression in the heart itself seems to be rather important for these observations. Smyrnias et al. observed in phenylephrine-treated cardiomyocytes an upregulation of nuclear factor erythroid-derived 2-like 2 (Nrf2), which depends on Nox4 (53). We observed increased beta-1 adrenergic receptor mRNA expression in the heart tissue from Nox4^−/−^ mice. This increase in beta-1 adrenergic receptor may represent a compensatory response to reduced catecholamines, as the heart tries to maintain adrenergic sensitivity despite lower norepinephrine levels. Both beta-1 and beta-2 adrenergic receptors respond to epinephrine and norepinephrine, with norepinephrine being 10 times more selective for the beta-1 adrenergic receptor (54).

To test the impaired vascular reactivity and impaired ability to adapt to changes in blood pressure, we used a model of voluntary running devices. Physical activity increases the release of catecholamines, including norepinephrine, from the adrenal medulla, enhancing cardiovascular and metabolic responses to meet increased energy demands (28). Already, moderate-intensity exercise leads to increased peripheral catecholamine concentrations in humans (55). We found that the Nox4-depleted mice had lower norepinephrine concentrations in the urine compared with WT mice, also after the 20-week period of physical activity, further explaining the diminished exercise performance of Nox4^−/−^ mice previously reported by us (16). In addition to the effect on norepinephrine concentration, we found smaller adrenal glands in the Nox4^−/−^ mice after 20 weeks of access to voluntary running devices. Bartalucci et al. reported an increase in adrenal gland size of 31% for mice undergoing high-intensity exercise and 10% for those undergoing low-intensity exercise. This enlargement was attributed to an increase in both the adrenal cortex and medulla areas (56), which was absent in the Nox4^−/−^ mice. In humans, exercise is also known to stimulate the hypothalamic–pituitary–adrenal axis, which increases adrenocorticotropic hormone (ACTH) and subsequently cortisol release from the adrenal cortex (57). Therefore, analyzing the impact of the adrenal cortex and glucocorticoids in this context in our model would be of importance in future studies. While we did not observe differences in cardiac performance between WT and Nox4^−/−^ mice having access to voluntary running devices, in a model of forced exercise, Hancock et al. detected cardiac dysfunction leading to reduced exercise capacity (29). Thus, the magnitude of the effects resulting from Nox4 loss may depend on the level of acute stress and the associated catecholamine response.

From a translational perspective, it will be important to determine whether Nox4-dependent redox signaling contributes more broadly to noradrenergic and sympathetic regulation within the central nervous system. Such studies may provide further insights into the relevance of Nox4-mediated norepinephrine regulation to disorders associated with altered sympathetic and noradrenergic signaling.

## 5 Conclusions

Nox4 plays a role in regulating norepinephrine synthesis and release in the adrenal medulla through its H_2_O_2_-producing activity. Thereby, Nox4 ensures proper norepinephrine levels and an adequate vascular response. Understanding Nox4’s precise functions might offer potential therapeutic targets for adrenal and stress-related disorders.

## 6 Limitations

In this study, the effects of decreased norepinephrine concentrations in Nox4^−/−^ mice were detected, and functional consequences in these mice were described. We focused on vascular and cardiac effects, although we were aware that the scope of actions of norepinephrine extends far beyond that. Possible areas that could be affected include sympathetic neuron function, glycogenolysis, and lipolysis. In addition, in our model, we cannot rule out the possibility that norepinephrine’s ability to relax airway smooth muscles, thereby improving ventilation and oxygen uptake during exercise, is affected. Finally, the role of norepinephrine and its alpha-adrenergic receptors in neuronal function is implicated in several neurological diseases and should be the focus of further studies in our model (58). We consider adrenal endothelial cells to represent the major source of Nox4 in the adrenal gland. However, definitive confirmation at the protein level and of its cell-specific localization was not possible because of the limited specificity of available Nox4 antibodies.

## Data Availability

The raw data supporting the conclusions of this article will be made available by the authors, without undue reservation.
