# Supplementary material for: NADPH oxidase 4 as a source of hydrogen peroxide can regulate norepinephrine levels and vascular contraction
Source: Front Endocrinol (Lausanne). 2026 Oct 2;17:1932927. doi: 10.3389/fendo.2026.1932927 (PMC13671950; doi:10.3389/fendo.2026.1932927)
Supplement: Supplementary file 1 [file SupplementaryFile1.docx]

Supplementary Material

**NADPH oxidase 4 as a source of hydrogen peroxide can regulate norepinephrine levels and vascular contraction**

**Heike Brendel^1^***, **Jennifer Mittag^1^**, **Nicole Bechmann^2^, Amna Shahid Mehmood-Ashraf^1^, Antje Schauer^3^, Patrick Diaba-Nuhoho^1^, Mirko Peitzsch^2^, Graeme Eisenhofer^4^, Stefan R. Bornstein^4,5^, Henning Morawietz^1^*, and Coy Brunssen^1^**

^1^	Division of Vascular Endothelium and Microcirculation, Department of Medicine III, Faculty of Medicine and University Hospital Carl Gustav Carus at TUD Dresden University of Technology, Dresden, Germany; jennifer.mittag@ukdd.de (J.M.); aamabcn@gmail.com (A.S.); [Patrick.Diaba-Nuhoho@ukdd.de](mailto:Patrick.Diaba-Nuhoho@ukdd.de) (P.D.-N.); coy.brunssen@ukdd.de (C.B.)

^2^	Institute for Clinical Chemistry and Laboratory Medicine, University Hospital Carl Gustav Carus, Faculty of Medicine and University Hospital Carl Gustav Carus at TUD Dresden University of Technology, Dresden, Germany; nicole.bechmann@uniklinikum-dresden.de (N.B.); mirko.peitzsch@uniklinikum-dresden.de (M.P.)

^3^	Department of Internal Medicine and Cardiology, Heart Center Dresden - Laboratory of Experimental and Molecular Cardiology, Technische Universität Dresden, Dresden, Germany; antje.schauer@tu-dresden.de (A.Sch.)

^4^	Department of Medicine III, Faculty of Medicine and University Hospital Carl Gustav Carus at TUD Dresden University of Technology, Dresden, Germany; graeme.eisenhofer@uniklinikum-dresden.de (G.E.); stefan.bornstein@uniklinikum-dresden.de (S.R.B)

^5^	Department of Environmental and Public Health, College of Health Sciences, Abu Dhabi University, Abu Dhabi, United Arab Emirates

*	Correspondence: henning.morawietz@tu-dresden.de (H.M.); heike.brendel@uniklinikum-dresden.de (H.B.); Tel: +49-351-4586678 (H.B.)

**Supplementary Figure 1:** **Impact of increasing hydrogen peroxide concentrations on catecholamine release of PC12 cells. (A)** DOPA concentration, **(B)** dopamine concentration and **(C)** norepinephrine concentration in supernatant of control PC12 cells and cells treated with 10 µM H_2_O_2_ (n=8) or 50 µM H_2_O_2_ (n=4-5). Statistics: one-way ANOVA followed by Tukey’s multiple comparisons tests. * *p* < 0.05; ** *p* < 0.01. Abbreviations: DA, dopamine; DOPA, dihydroxyphenylalanine; NE, norepinephrine.

***Myocardial performance was similar in physically active WT and Nox4^-/-^ mice***

Physically active mice were examined using echocardiography (**Supplementary Figure 2A**). The myocardial performance were indicated by left ventricular ejection fraction (LVEF) (**Supplementary Figure 2B**), left ventricular fractional shortening (LVFS) (**Supplementary Figure 2C**), left ventricular end-diastolic volume (LVEDV) (**Supplementary Figure 2D**), left ventricular end-systolic volume (LVESV) (**Supplementary Figure 2E**), ratio of early mitral inflow and late mitral inflow (E/A) (**Supplementary Figure 2F**), ratio of early mitral inflow and tissue doppler mitral annulus velocity in early diastole (E/e’) **(Supplementary Figure 1G**), end-diastolic left ventricular inner diameter (LVID;d) (**Supplementary Figure 1H**), end-diastolic left ventricular posterior wall thickness (LVPW;d) (**Supplementary Figure 1I**), and end-diastolic left ventricular anterior wall thickness (LVAW;d) (**Supplementary Figure 1J**) in hearts of WT and Nox4^-/-^mice after 20 weeks voluntary running. In all parameter’s hearts of WT and Nox4^-/-^ mice performed similar.

**Supplementary Figure 2:** **Myocardial performance is similar in physical active WT and Nox4^-/-^ mice. (A)** Echocardiography in WT and Nox4^-/-^ mice after 20 weeks voluntary running. **(B)** Left ventricular ejection fraction (LVEF), **(C)** left ventricular fractional shortening (LVFS), **(D)** left ventricular end-diastolic volume (LVEDV), **(E)** left ventricular end-systolic volume (LVESV), **(F)** ratio of early mitral inflow to late mitral inflow (E/A), **(G)** ratio of early mitral inflow to tissue doppler mitral annulus velocity in early diastole (E/e’), **(H)** end-diastolic left ventricular inner diameter (LVID;d), **(I)** end-diastolic left ventricular posterior wall thickness (LVPW;d), and **(J)** end-diastolic left ventricular anterior wall thickness (LVAW;d) in hearts of WT and Nox4^-/-^mice after 20 weeks voluntary running (n≥8). Statistics: t test; * *p* < 0.05.
